# PDMS-PMOXA-Nanoparticles Featuring a Cathepsin B-Triggered Release Mechanism

**DOI:** 10.3390/ma12172836

**Published:** 2019-09-03

**Authors:** Daniel Ehrsam, Fabiola Porta, Janine Hussner, Isabell Seibert, Henriette E Meyer zu Schwabedissen

**Affiliations:** Department Pharmaceutical Sciences, University of Basel, Klingelbergstrasse 50, 4056 Basel, Switzerland

**Keywords:** enzyme-triggered-release, cathepsin B, paclitaxel, nanoparticles, PDMS-PMOXA, cancer, ovarian cancer

## Abstract

Background: It was our intention to develop cathepsin B-sensitive nanoparticles for tumor-site-directed release. These nanoparticles should be able to release their payload as close to the tumor site with a decrease of off-target effects in mind. Cathepsin B, a lysosomal cysteine protease, is associated with premalignant lesions and invasive stages of cancer. Previous studies have shown cathepsin B in lysosomes and in the extracellular matrix. Therefore, this enzyme qualifies as a trigger for such an approach. Methods: Poly(dimethylsiloxane)-b-poly(methyloxazoline) (PDMS-PMOXA) nanoparticles loaded with paclitaxel were formed by a thin-film technique and standard coupling reactions were used for surface modifications. Despite the controlled release mechanism, the physical properties of the herein created nanoparticles were described. To characterize potential in vitro model systems, quantitative polymerase chain reaction and common bioanalytical methods were employed. Conclusions: Stable paclitaxel-loaded nanoparticles with cathepsin B digestible peptide were formed and tested on the ovarian cancer cell line OVCAR-3. These nanoparticles exerted a pharmacological effect on the tumor cells suggesting a release of the payload.

## 1. Introduction

According to the World Health Organization, cancer is one of the leading causes of morbidity and mortality worldwide, and therefore, cancer therapy is an important objective for today’s medicinal research [1]. Different therapeutic approaches have been taken so far, from surgical removal to radio- and chemotherapy. The use of anti-proliferative drugs in cancer therapy means that every part of the human body can be reached and, thus, not only the primary cancer can be fought, but also metastasized cells [2]. For most compounds used in chemotherapy though, the mode of action is not exclusive to cancer cells but affects cell proliferation throughout the entire organism. This leads to adverse side effects and limits the doses that can be applied to patients [3,4]. Current efforts in chemotherapeutic drug development aim at increasing the selectivity for cancer cells while reducing the systemic exposure.

Many strategies have been applied to direct small molecules to the target cells. These include drug delivery systems like nanoparticles. Nanoparticles are 3-dimensional supramolecular entities, which are assumed to passively accumulate in the tumor tissue due to the enhanced permeability and retention effect [5]. In detail, macromolecules including nanoparticles are passively enriched in neovascularized tumor tissue as the newly formed blood vessels exhibit not only enhanced perfusion, but also increased permeability. In addition, the lymphatic system in tumors is assumed to be less effective, which is leading to decreased lymphatic drainage from the tumor site. Although the enhanced permeability and retention effect (EPR) is a very interesting phenomenon, it is also assumed to be heterogeneous in humans [6].

Hitherto, a variety of nanoparticles using many different materials has been developed, investigated and some have been approved for the use in humans [7,8]. Since 1995, the United States Food and Drug Administration (FDA) approved over 50 nanopharmaceuticals; the majority being liposomal, nanocrystal, or polymeric formulations. The latter are comprised of amphiphilic polymers consisting of subunits also known as block copolymers. In detail, linear block copolymers can be divided into the categories of diblock copolymers or of triblock copolymers. Diblock copolymers possess a polar and a non-polar homopolymeric subunit (A-B). Triblock copolymers consist of three homopolymeric subunits (A-B-A or A-B-C), where two subunits can be similar [9]. Nanoparticles formed by block copolymers are known as polymersomes or polymeric nanoparticles depending on the membrane.

An entity of polymersomes, investigated for their application as a drug delivery system, are PDMS-PMOXA polymersomes where the diblock copolymer used for the formulation is poly(dimethyl siloxane)-poly-*b*-(methyloxazoline). So far, these polymersomes were investigated for their toxicological and biocompatibility profile, showing no significant toxicity in in vitro models [10,11] and the subunits have been reported to be biocompatible [12]. A previous study has described that the hydrophilic PMOXA-block is cleared by the kidney from the circulation [13]. Although the PDMS-block is hydrophobic, as long as the molecular weight does not surpass 5 kDa, it undergoes renal elimination [14]. Importantly, polymersomes have the ability to encapsulate bioactive molecules like chemotherapeutics [7], and are therefore, suitable for drug delivery.

Most of the current approved nanoformulations are aiming at improving the pharmacokinetics and pharmacodynamics by packaging/conjugating drugs in/to nanoparticles [15]. However, incorporating an active or triggered release strategy could lead to a dose reduction and therefore, decrease side effects [16,17]. A mechanism that could be utilized for such an approach is triggered release of the payload using tumor-specific elicitors [18]. Here, the tumor-associated expression of enzymes in or close to the targeted tissue offers potential candidates. Especially, enzymes exerting proteolytic activity could be employed to cleave drug–peptide conjugates or to trigger changes in the drug carrier’s outer layer [16]. An enzyme qualifying as an elicitor is cathepsin B.

In physiological conditions, cathepsin B is located in the lysosomes [19], where it is involved in the degradation and, therefore, regulation of proteins. Besides proteolysis within the lysosome, cathepsin B is involved in cell death mediation [20], and it contributes to the degradation of the extracellular matrix [21]. Indeed, in cancer, cathepsin B is secreted by tumor cells where it contributes to the degradation and remodeling of the extracellular matrix and whereby facilitating tumor cell invasion into the surrounding tissue [22]. In tumor tissue, cathepsin B seems to be expressed predominantly in areas bordering the extracellular matrix [23]. By analyzing patient samples, increased protein content or activity of cathepsin B have been detected in ovarian [19] and colorectal [24] cancer. A similar enhancement has been observed in a B16 mouse melanoma in vivo model [25]. Jedeszko et al. summarized the core-statements of many publications in a review showing increased expression and activity of cathepsin B in breast, colon, lung, prostate cancer, glioblastoma, and melanoma [26].

In this study, we aimed at using cathepsin B as an elicitor for enzyme-triggered drug release from PDMS-PMOXA-based nanoparticles. The PDMS-PMOXA nanoparticles were used as a platform for surface-modifications. The separate surface-modification steps were surveyed by Fourier-transform infrared (FT-IR) spectroscopy. Because cathepsin B (CTBS) plays a pivotal role in the release mechanism, its mRNA expression was determined in patient derived tumor samples. Accordingly, a suitable in vitro cell model system was identified and subsequently used for in vitro studies on the pharmacological activity of the CTSB-degradable nanoparticles (Supplemental Tables S1 and S2).

## 2. Materials and Methods

### 2.1. Materials

Poly(dimethylsiloxane)-b-poly(methyloxazoline) PDMS-PMOXA was purchased from Polymer Source Inc., Ottawa, Canada. Organic solvents were obtained from J.T. Baker, (Deventer, Netherlands), Carl Roth GmbH + Co. KG (Arlesheim, Switzerland), or from Sigma Aldrich (Buchs, Switzerland). p-Maleidoimino phenylisocyanate was purchased from Invitrogen (distributed by Thermo Fisher, Reinach, Switzerland). Double distilled water (ddH_2_O) with a resistivity of 18.2 mΩ was generated with a Barnstead Nanopure DiamondTM System (Thermo Fischer).

### 2.2. Quantitative PCR for Gene Expression Analysis in Cancer Tissue Samples and Representative Cell Lines

To determine CTSB mRNA expression in cancer tissue a cDNA array (CSRT103, Origene, Rockville, MD, USA) was commercially obtained. Furthermore, mRNA of cancer cell lines was isolated using peqGOLD RNA pure (Axon Lab, Baden, Switzerland) and reverse transcribed using the High-Capacity cDNA Reverse Transcription kit (Thermo Fisher). The amount of CTSB was assessed by quantitative real-time PCR (qPCR) using the pre-developed TaqMan^TM^ assays (Thermo Fischer) Hs00947439_m1, the TaqMan^®^ gene expression mastermix, and the ViiA^TM^ 7 Real-Time PCR System. The reaction was carried out in a volume of 15 µL composed of 0.75 µL of the Hs00947439_m1-FAM TaqMan assay, 6.75 µL H_2_O, and 7.5 µL TaqMan^®^ Gene Expression Mastermix (Applied Biosystems, LubioSciences, Lucerne, Switzerland). For quantification of copy numbers, a standard curve using cloned PCR amplicon was recorded.

### 2.3. Immunohistochemical Staining of CTSB in Human Ovary Cancer Tissue

For the detection of CTSB by immunohistochemical staining a commercially obtained array of paraffin embedded tissue sections was used. This array included malignant transformed and non-malignant transformed samples of various tissues (MTU951, BioCat GmbH, Heidelberg, Germany). To deparaffinize the tissue sections, two changes of xylol and rehydration in a decreasing ethanol series ranging from 96% to 0% was executed, followed by a heat induced epitope retrieval in 0.1 M citrate buffer (pH = 6.0, 20 min). The endogenous peroxidase was quenched in a 3%-H_2_O_2_-methanol-bath for 20 min. To reduce unspecific binding of the antibody, the slides were exposed for 1 h to blocking solution (5% FCS and 1% BSA in PBS). CTSB was detected with the primary anti-CTSB-antibody (sc-13985, Santa Cruz Biotechnology, Inc., Texas, TX, USA) at a dilution of 1:1000 in blocking solution. After incubation with primary antibody overnight at 4 °C, the tissue slides were washed repeatedly in PBS. The tissue slides were then exposed to the secondary HRP-coupled goat-anti-rabbit antibody (Bio-Rad Laboratories Laboratories, Cressier, Switzerland; 1:100) for two hours at room temperature (RT). After several washing steps in PBS, 1 mg/mL of diaminobenzidine (DAB) diluted in 0.05 M phosphate buffer containing 0.02% H_2_O_2_ was added for visualization of epitope-bound antibody. Nuclei were stained with hematoxylin solution (Carl Roth GmbH + Co. KG) and slides were mounted with Roti^®^-Histokitt II (Carl Roth GmbH + Co. KG). Finally, the stained tissue slides were imaged with a Leica DMi8 microscope equipped with a DFC 365 FX camera (Leica, Heerbrugg, Switzerland) and the LAS software Version 4.6 (Leica).

### 2.4. Western Blot Analysis

Protein samples were collected from cultured cells seeded at a density of 1 × 10^6^ cells/ 10 cm dish. After reaching 80% confluence, the culture medium was replaced by the respective medium containing no FCS to avoid contamination with serum proteins. After 24 h, the culture supernatant was collected to enrich the secreted protein. In detail, the supernatant was supplemented with ice-cold 10% trichloroacetic acid, kept on ice for 15 min, and was then centrifuged for 20 min at 17,000 × *g* and 4 °C. The precipitate was washed twice with ice-cold acetone (5 mL), and was then air-dried for 30 min at room temperature. The enriched secreted proteins were finally solubilized in 6 M urea. To determine the protein content, the Bradford assay (Thermo Fischer) was used. Cell lysate was collected harvesting the cells in 5 mM Tris-HCL supplemented with protease inhibitor cocktail (Sigma Aldrich, Buchs, Switzerland), followed by three cycles of freezing thawing in liquid nitrogen. For Western blot analysis protein samples were supplemented with Laemmli and then separated by a 10% SDS-PAGE. Afterwards, the proteins were electrotransferred to a nitrocellulose membrane (Bio-Rad Laboratories). After blocking with 5% FCS/1% albumin in TBS-T, the membranes were exposed overnight and at 4 °C to the primary antibody sc-365558 (Santa Cruz Biotechnology, diluted 1:500) for cathepsin B or sc-47778 (Santa Cruz Biotechnology, diluted 1:1000) for actin. Thereafter, the blot was exposed to the respective HRP-conjugated secondary antibodies (Bio-Rad Laboratories) for 1 h at RT. Pierce™ ECL Western Blotting Substrate (Thermo Fisher) and the ChemiDoc™ MP Imaging System equipped with the image lab software (version 4.1) both from Bio-Rad Laboratories were used for image acquisition.

### 2.5. Detection of CTSB Activity by Enzymatic Assay

CTSB’s activity in OVCAR-3 and OVCAR-5 cells was detected by the liberation of the fluorescent 7-amino-4-methylcoumarin from Z-Arg-Arg 7-amido-4-methylcoumarin (Sigma-Aldrich). The assay was performed according to the instruction manual and as previously described by Barrett et al. [27]. In short, 60 μL of 8 mM L-cysteine-HCl in 352 mM potassium phosphate buffer (including 48 mM sodium phosphate, and 4.0 mM ethylenediaminetetraacetic acid), 70 μL 0.1% Brij 35 solution in purified water, 10 μL of cell lysate or supernatant and 60 μL 0.02 mM of Nα-CBZ-Arg-Arg-7-amido-4-methylcoumarin in 0.1% Brij 35 solution. The release of 7-amino-4-methylcoumarin was measured with the microplate reader Tecan Infinite M200 Pro (Tecan, Männedorf, Switzerland; excitation = 348 nm, emission = 440 nm).

### 2.6. Cell Culture

The cell lines OVAR-5 (RRID:CVCL_1628), and OVCAR-3 (ATCC HTB-161) were commercially obtained from the American Tissue Culture collection, (Manassas, MA, USA). OVCAR-3 were cultured in RPMI-1640 (BioConcept) supplemented with 20% FCS, 1% non-essential amino acids (MEM-NEAA, BioConcept), and 1% GlutaMAX. OVCAR-5 were cultured in DMEM supplemented with (v/v) 10% FCS, 1% MEM-NEAA, and 1% GlutaMAX. In viability assays, 1% Penicillin/Streptomycin (BioConept) was added to the media. All cell lines were kept at 37 °C in a humidified atmosphere with 5% CO_2_.

### 2.7. Immunofluorescence of Cathepsin B in Ovarian Cancer Cells

OVCAR-5 and OVCAR-3 cells were seeded at a density of 2 × 10^5^ cells/well on cover slips placed in 12 well plates. One day after seeding, cells were fixed with ice-cold methanol-acetone (1:1, v/v) for 5 min at −20 °C. After several washing steps with PBS, unspecific antibody binding was prevented by incubation with 5% normal goat-serum/ 0.3% Triton X-100 in PBS for 1 h at room temperature. Cells were incubated with the primary antibody anti-Cathepsin B (sc-366558, Santa Cruz Biotechnology Inc., Dallas, Texas, TX, USA) diluted in antibody dilution buffer (1% BSA/0.3% Triton X-100 in PBS, 1:10) overnight at 4 °C. After washing with PBS, cells were incubated with the secondary Alexa Fluor™ goat-anti-mouse 568 antibody (Thermo Fisher) diluted in antibody dilution buffer (1:200). After incubation for 1 h at room temperature, cells were washed with PBS and mounted using Roti^®^-Mount FluorCare containing DAPI for nuclei stain (Carl Roth GmbH + Co. KG). For antibody control, the primary antibody was omitted. Images were taken with the Leica DMi8 Microscope with the MC 170HD camera and LASV4.8 software (Leica Microsystems, Heerbrugg, Switzerland).

### 2.8. Assessment of Hydrodynamic Radius and Surface Charge of the Nanoparticles

To measure the hydrodynamic radius of the nanoparticles, the dynamic light scattering (DLS) technique was used (Malvern Zetasizer NanoSeries, Malvern Instruments GmbH, Herrenberg, Germany). Briefly, the samples were degassed utilizing a Thermo Vac sample degassing and thermostat system (MicroCal^TM^, Malvern Instruments GmbH). The samples were measured using a backscattering angle of 173° was used. Data were analyzed using the Zetasizer software 7.11. (Malvern).

### 2.9. Critical Aggregation Concentration of PDMS-PMOXA

Critical aggregation concentration (CAC) was determined by utilizing the different fluorescence characteristics of pyrene (Sigma-Aldrich) in a hydrophobic or hydrophilic environment [28,29]. Briefly, 500 μL 2.4 μM pyrene in aceton and corresponding amount of PDMS-PMOXA (2500, 250, 25, 2.5, 0.25, 0.025 μg) in acetone were added to a glass vial. The solvent was evaporated for 1.5 h at 40°C and under constant flow of nitrogen. After completely drying the pyrene and PDMS-PMOXA, 1 mL of ultrapure water was added, and particles were formed by ultrasonification. The fluorescence of pyrene was measured with the microplate reader Tecan Infinite M200 Pro (Tecan; excitation 332 nm, emission I_1_ = 373 nm and I_3_ = 384 nm). To determine the CAC, the pyrene’s intensity ratio of I_1_/I_3_ was plotted against the logarithm of the PDMS-PMOXA concentration.

### 2.10. Synthesis of Carboxyl Terminated Poly(Dimethylsiloxane)-b-Poly(Methyloxazoline)

Carboxyl terminated poly(dimethylsiloxane)-b-poly(methyloxazoline) (PDMS-PMOXA) was synthesized as described before [30]. Briefly, 15.6 µmol (101.40 mg) of PDMS-PMOXA (PDMS_67_-*b*-PMOXA_15_, Mn = 5000-b-1300, polydispersity index PDI = 1.25) was solved in 5 mL of dichloromethane (DCM). 87.4 µmol (8.75 mg) of succinic anhydride, 15.6 µmol (1.91 mg) of dimethyl aminopyridine (DMAP, Sigma Aldrich), and 79.5 µmol (11 µL) of triethylamine (TEA, Sigma Aldrich) were added to the reaction. The reaction was carried out overnight slowly reaching room temperature. An ultrafiltration using a dialysis membrane (MWCO = 1000, Spectrapor, Spectrum labs, Breda, Netherlands) for 24 h was carried out to purify the reaction. Fourier transformed infrared (FT-IR) spectroscopy was performed to confirm the final compound structure.

### 2.11. Synthesis of N-Hydroxisuccinimide Activated Poly(Dimethylsiloxane)-b-Poly(Methyloxazoline)

After dissolving the carbonyl terminated poly(dimethylsiloxane)-b-poly(methyloxazoline) in 5 mL of dichloromethane in a round bottom flask, the reaction mixture was cooled to 4 °C. Then, 65.4 µmol (10.2 mg) of N-(3-dimethylaminopropyl)-N′-ethylcarbodiimide hydrochloride (EDAC, Sigma Aldrich) and 100 µmol (11.5 mg) of N-hydroxylsuccinimide (NHS, Sigma Aldrich) were added to the mixture. The final compound was recovered after performing dialysis for 24 h in DCM. The N-hydroxisuccinimide activated poly(dimethylsiloxane)-b-poly(methyloxazoline) was obtained in quantitative yield and FT-IR analysis was performed to confirm the final structure.

### 2.12. Surface Modification of Polymer Vesicles with the NH2-Ahx-GSGFLGSC Peptide and Paclitaxel Loading

Nanoparticles holding a paclitaxel payload were formulated as described before [30]. The nanoparticles were diluted five times (200 µL nanoparticle suspension in 800 µL PBS) leading to a 1 mg/mL of nanoparticle suspension. To 1 mL of 1 mg/mL of nanoparticle suspension, 10 µL of 4.7 mM p-maleimidophenyl isocyanate (PMPI) in dimethyl sulfoxide (DMSO) were added and stirred overnight at room temperature to allow modification of the nanoparticle surface. The peptide Fmoc-Ahx-GSGFLGSC (GSG; Ahx = aminocaproic acid; Biomatik, Cambridge, Ontario, Canada) was deprotected as described before [30]. In brief, 76 µL of piperidine were added to 10 mg of Fmoc-protected GSG-peptide in 300 µL dimethylformamide and stirred for 1 h at room temperature. A cold ether precipitation was conducted to recover the deprotected GSG-peptide. Ten µL of a 10 mg/mL GSG-peptide solution in PBS and 10 µL EDAC (10 mg/mL in MilliQ water) were added to the 1mg/mL solution of nanoparticles to react overnight at room temperature. After coupling the peptide to the nanoparticle’s surface, an additional extrusion similar to the previously described was performed. The nanoparticles were then purified by ultrafiltration (MWCO 12-14 kDa, Spectrapor) overnight at room temperature against PBS.

### 2.13. Determination of the Encapsulation Efficiency.

To determine the encapsulation efficiency, high-performance liquid chromatography (HPLC) analysis was used. The GSG-modified polymer membrane was disrupted by the use of Triton-X (Merck, Zug, Switzerland) in water and acetonitrile (VWR International, Dietikon, Switzerland). In detail, 25 µL of 1 mg/mL nanoparticle (theoretically containing 0.2 mg/mL paclitaxel), 25 µL of 0.4% triton-X in water and 50 µL acetonitrile were combined, vortexed and centrifuged for 3 min at 4000 × *g*. The amount of paclitaxel encapsulated in the nanoparticles was detected with an Agilent 1100-series equipped with diode array and evaporating light scattering detector (Agilent Technologies, Basel, Switzerland). The mobile phase consisted of ddH_2_O (buffer A) and acetonitrile (buffer B, VWR International, Dietikon, Switzerland). Separation was achieved with a Poroshell C18 column (3.0 × 100 mm 2.7-micron, Agilent Technologies). Starting at 50% A, 50% B changing from minute 1 to minute 11 to 0% A, 100% B, with a flow rate of 0.45 mL/min. The paclitaxel peak had a retention time of 4.8/4.9 min. To calculate the concentration of encapsulated paclitaxel a paclitaxel standard curve (Cathepsin B Table S3A,B; AUC versus concentration) was recorded.

### 2.14. Fluorescent Labeling of the Polymeric Nanoparticles

Fluorescent labeling of the polymeric nanoparticles was achieved by incorporating 3,3′-dioctadecyloxacarbocianine perchlorate (DiO, Sigma-Aldrich). In brief, 5 μL of a 25 mg/mL DiO stock solution in ethanol was added to the DCM during preparation of the thin-film layer. The dried thin-film layer was then rehydrated with PBS as described above.

### 2.15. Release and Uptake of Fluorescently Labeled Nanoparticles

The fluorescently labeled nanoparticles were bound to a Pierce™ maleimide activated black 96-well-plate (Thermo Fisher) according to the manufacturer’s manual. In brief, after several washes with washing buffer, 100 µL of a fluorescently-labeled polymeric nanoparticle-solution (200 µg/mL in binding buffer) were added to each well and incubated over night at 4 °C to achieve nanoparticle binding to the plate’s surface. The surface binding was followed by additional washes with washing buffer. Not reacted maleimide-groups were deactivated by incubation with 100 µL 10 mg/mL L-cysteine-solution for 1 h at RT. After several washes, 100 µL of 100 µg/mL cathepsin B, 100 µg/mL cathepsin B supplemented with 100 µM CA-074, or PBS solvent control were added, respectively. After incubation for 24 h at 37 °C, the difference in fluorescent signal on the 96-well without supernatant was measures using the microplate reader Tecan Infinite M200 Pro (excitation = 490 nm, emission = 530 nm). The reduction in fluorescent signal measured on the plate’s surface was interpreted as evidence of release caused by cathepsin B.

### 2.16. Cell Viability Assay

OVCAR-3 cells were seeded in 96-well plates at a density of 15,000 cells/well to assess cell viability. To determine the impact of PDMS-PMOXA-GSG-Paclitaxel particles on cell viability, cells were treated one day after seeding with increasing concentrations of paclitaxel (0.01 nM to 1 µM). The content of paclitaxel in the nanoparticles was quantified by HPLC. In order to analyze whether the release of paclitaxel is mediated by cellular cathepsin B, cells were pre-treated with 0.1 µM of the cathepsin B inhibitor CA-074 (Sigma-Aldrich) or DMSO control three hours after seeding. After 24 h, cells were exposed to PDMS-PMOXA-GSG-Paclitaxel with or without 2 μM CA-074. As positive control, cells were incubated with 25 µg/mL cathepsin B from human placenta (Sigma-Aldrich). After 48 h, cell viability was measured using the Fluorometric Cell Viability Kit I (Resazurin) from PromoKine (Vitaris AG, Baar, Switzerland). The microplate reader Tecan Infinite M200 Pro (Tecan) was used for quantification of cell viability (fluorescence, excitation = 530 nm, emission = 590 nm). Data are presented as mean ± SD as percent of control. IC_50_ values were estimated using a three-parameter logistic function assuming a standard slope.

### 2.17. Statistical Analysis

Statistical analysis was performed with the GraphPad prims software (version 6, GraphPad Software Inc. La Jolla, CA, USA). For statistical analysis of real time PCR a student’s t-test or one way ANOVA with multi-comparison was applied. Statistical analyses of cell viability studies were performed by column statistics with one-sample student’s t-test. In vitro data points mentioned in this publication consist of at least three independent experiments each performed with two biological replicates. A *p*-value ≤ 0.05 was considered statistically significant.

## 3. Results

### 3.1. Comparison of Cathepsin B in Tumor Entities of Female Organs.

With the intention to confirm presence of the peptidase in malignant transformed cells, the number of cathepsin B mRNA copies was determined in tumor entities originating from cervix, breast, endometrium, and ovary. Comparison of the number of transcripts in healthy and malignant transformed tissue revealed a statistically significant reduction of the amount of transcripts in tumors originating from breast compared to healthy tissue (mean number of CTSB copies ± SD; healthy vs. tumor 13,276 ± 3527 n = 2 vs. 2583 ± 3674 n = 23, Mann–Whitney test; *p* = 0.002). A similar trend was observed for the cervix (mean number of CTSB copies ± SD; healthy vs. tumor 15,502.80 ± 20,072 n = 4 vs. 5207.09 ± 3475 n = 9, Mann–Whitney test; *p* = 0.144), and the endometrium, whereas in tumors originating from ovary, the amount of transcripts did not exhibit this trend (healthy vs. tumor; 3685 ± 1318, n = 3 vs. 6922 ± 6782 n = 21; Mann–Whitney test; *p* = 0.172). Subsequently, we analyzed the data set on the cathepsin B mRNA expression for the impact of the tumor stage (Supplemental Figure S1). There was a statistically significant reduction in breast cancer stage I, II, and III compared to healthy breast tissue (Supplemental Figure S1A; mean copy numbers ± SD normal vs. stage I, II, and III; 13,276 ± 3527; 3593 ± 881.2; 3378 ± 2954; 3831 ± 5088), suggesting that reduction is independent of the tumor stage. No statistically significant differences in cathepsin B mRNA expression was detected comparing different tumor stages in samples deriving from cervix (Supplemental Figure S1B) or endometrium (Supplemental Figure S1C). Interestingly, stage I ovarian carcinoma showed a trend to increased expression of cathepsin B mRNA compared to normal tissue (Supplemental Figure S1D).

**Figure 1:**
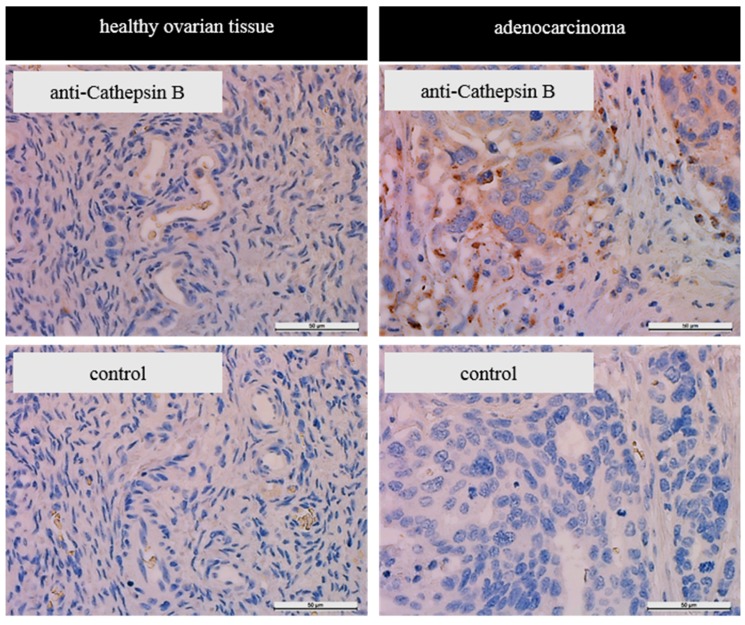
Cathepsin B expression in ovarian carcinoma and healthy tissue. Protein expression was detected by immunohistochemistry in healthy ovarian tissue or ovarian adenocarcinoma. In control sections, the primary antibody was omitted. Scale bar 50 µm.

To further validate the presence of cathepsin B in ovarian tumor tissue, immunohistochemistry of a healthy ovarian tissue, and an ovarian adenocarcinoma sample was performed. As shown in Figure 1, there was staining of only a limited number of cells in ovarian tissue. In the adenocarcinoma tissue a disperse coloring in the surrounding of the cells was observed, with strong staining in some cells. In cells, CTSB appeared to be localized in intracellular granules. To validate our results assessed on a limited sample size, a literature search was conducted. Six previous publications were found describing increased CTSB expression, content or activity in ovarian cancer compared to healthy tissue (Supplemental Table S2).

### 3.2. Characterization of CTSB Expression in Human Ovarian Cancer Cell Lines.

**Figure 2:**
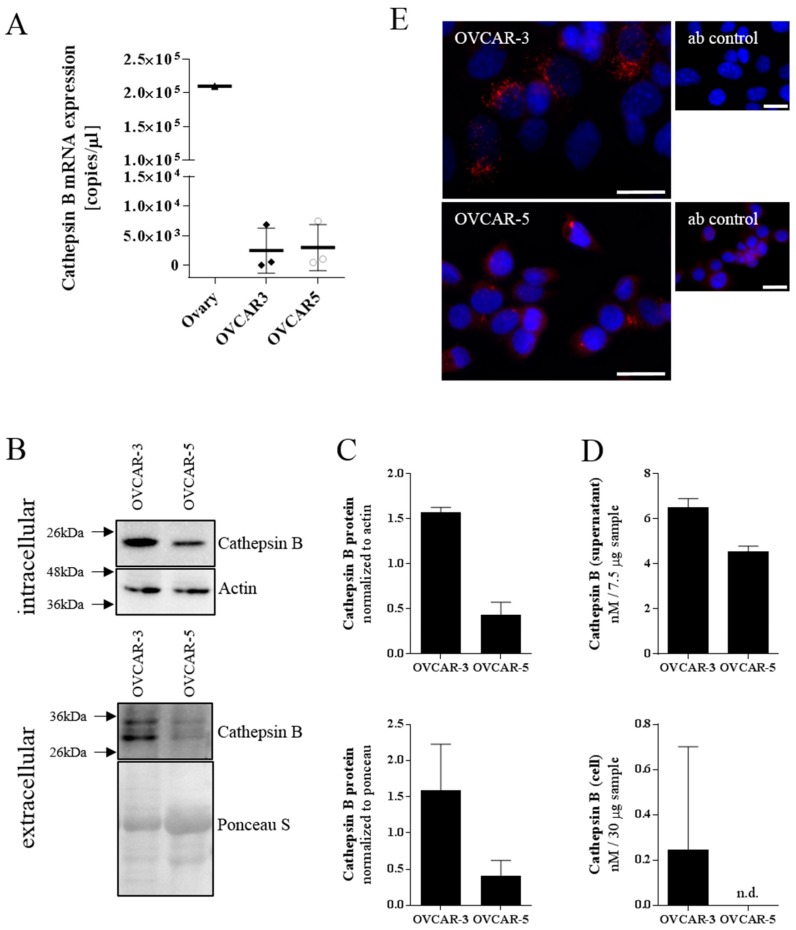
Cathepsin B expression in ovarian cancer cell lines. (**A**) mRNA expression of cathepsin B assessed by qPCR. (**B**) Western blot analysis of cathepsin B, protein loading and actin in cell supernatant and in the cytoplasm of different cell lines. (**C**) Cathepsin B signal from cell supernatant normalized to ponceau S and intracellular cathepsin B signal normalized to actin signal. (**D**) Activity of cathepsin B inside the cells and in the cell supernatant. (**E**) Immunofluorometric microscopy of cathepsin B (red) and nuclei (blue) in ovarian cancer cell lines. Scale bar 25 µm.

In order to test the herein described cathepsin B degradable nanoparticles in vitro, we characterized two different ovarian cancer cell lines for the expression of this enzyme in comparison to human ovary. In these two ovarian carcinoma cell lines, namely OVCAR-3 and OVCAR-5, the mRNA expression of cathepsin B was comparable (Figure 2A), even if lower than in the human tissue samples. However, Western blot analysis of the intra- and extracellular protein fraction suggested a much higher amount of cathepsin B in OVCAR-3 compared to OVCAR-5 (Figure 2B). This was even more evident after normalization of the cathepsin B band to that of actin (Figure 2C; mean normalized protein amount ± SD; OVCAR-3 vs. OVCAR-5; intracellular: 1.568 ± 0.0580 vs. 0.4325 ± 0.1415; unpaired t-test; *p* = 0.0002; n = 3; extracellular: OVCAR-3 vs OVCAR-5; 1.587 ± 0.6411 vs. 0.4069 ± 0.2154). Assessing CTSB activity by liberation of 7-amino-4-methylcoumarin from Z-Arg-Arg-7-amido-4-methylcoumarin showed increased turnover in presence of the cell lysate of OVCAR-3 compared to OVCAR-5 (Figure 2D; mean nM of CTSB in 75μg intracellular protein ± SD; OVCAR-3 vs. OVCAR-5; 6.507 ± 0.3852 vs. 4.539 ± 0.2410; unpaired t-test; *p* < 0.0001; n = 3). Similar results were obtained for the extracellular protein fraction (mean nM of CTSB in 100 μg extracellular protein ± SD; OVCAR-3 vs. OVCAR-5; 0.2457 ± 0.4563 vs. not detectable; n = 3), where no CTSB activity was detected in the supernatant of OVCAR-5 cells. Our finding was further confirmed by immunofluorescent staining detecting cathepsin B in the cells. As shown in Figure 2E the staining of cathepsin B was observed in intracellular vesicles and was more intense in the OVCAR-3 cell line. Accordingly, we selected this cell model for further investigations of the herein described nanoparticles.

### 3.3. FT-IR Analysis of PDMS-PMOXA Modification Steps

**Figure 3:**
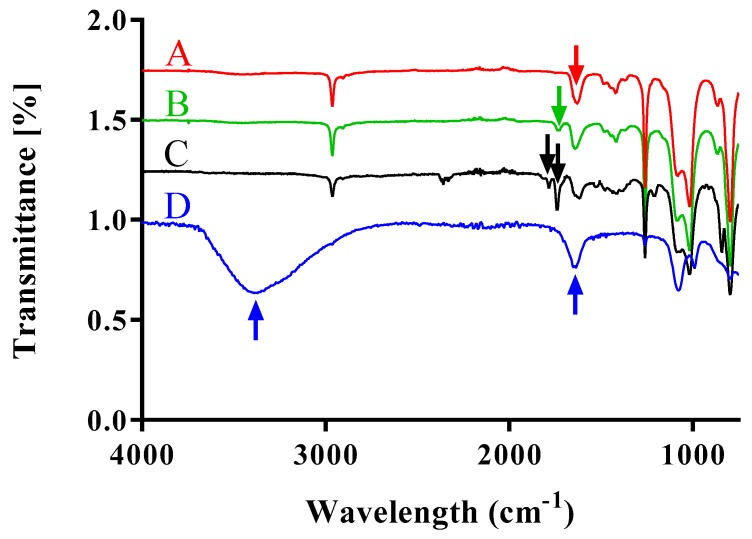
Fourier transform infrared spectra of modified poly(dimethylsiloxane)-poly(methyloxazoline). (**A**) FTIR spectrum of the diblock copolymer before modification. The arrow indicates a peak at 1690 cm^−1^, which corresponds to the carbonyl stretching of the PMOXA moieties. (**B**) FTIR spectrum of the carboxylic acid modified diblock copolymer. The arrow shows a stretching peak at 1732 cm^−1^ indicating the presence of the carbonyl group. (**C**) FTIR spectrum of the N-hydroxylsuccinimide (NHS) activated polymer. Here, the arrows indicate the peaks at 1738 and 1789 cm^−1^ which show the NHS modification of the polymer (carbonyl stretching in the COO-NHS ester moiety). (**D**) FTIR spectrum of GSG-modified nanoparticles. N–H stretching vibrations at 3368 cm^−1^ (Amide-A) are visible. C=O stretching vibrations (Amide I) peaks at 1635 cm^−1^.

The polymer was modified by standard coupling reactions and the Michael addition. Each synthetic step was characterized by FT-IR to assure the modification. The unmodified PDMS-PMOXA (Figure 3A) showed a distinct peak at 1690 cm^−1^ reflecting the carbonyl stretching. After modification of PDMS-PMOXA with succinic anhydride, an additional peak at 1732 cm^−1^ appeared (Figure 3B). After activation with NHS, additional peaks at 1738 cm^−1^ and 1789 cm^−1^ were visible representing the carbonyl stretching of the COO-NHS ester (Figure 3C). Finally, modification of the nanoparticle surface with the GSG-peptide caused peaks for N-H vibrations (amide-A) at 3368 cm^−1^ and C=O stretching (amide I) at 1635 cm^−1^ (Figure 3D).

### 3.4. Characterization of PDMS-PMOXA-GSG Nanoparticles

**Figure 4:**
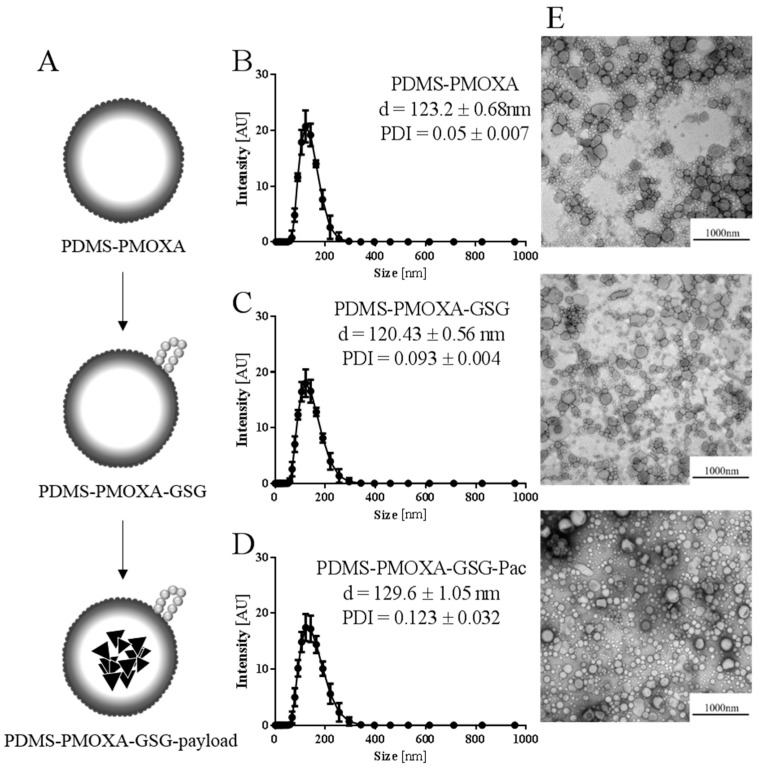
Characterization of nanoparticles by dynamic light scatting and electron microscopy. (**A**) The schematic representation shows the different stages at which diameter and shape was recorded. The impact of the surface-modification on hydrodynamic diameter and shape are illustrated for PDMS-PMOXA particles without surface-modification (**B**) surface-modified PDMS-PMOXA particles without payload (**C**) and surface-modified PDMS-PMOXA particles with paclitaxel payload (**D**) Data are represented as mean ± SD. (**E**) Transmission electron microscopy images reflected the size measured by DLS.

Diblock-copolymer PDMS-PMOXA nanoparticles loaded with paclitaxel were formulated using the thin-film technique followed by rehydration in PBS buffer. A series of extrusion was performed to homogenize the size distribution. Finally, the surface of the nanoparticles was cross-linked using the GSG-peptide. The different nanoparticles are schematically depicted in Figure 4A. Subsequently, the hydrodynamic diameter was assessed by dynamic light scattering comparing loaded and unloaded nanoparticles before and after peptide surface-modification. For the unloaded nanoparticles the surface-modification of the nanoparticles did influence the observed hydrodynamic diameter slightly but statistically significant (mean diameter ± SD, PDMS-PMOXA vs. PDMS-PMOXA-GSG; 123.2 ± 0.68 nm vs. 120.43 ± 0.56 nm; n = 3; one-way ANOVA, *p* < 0.05; Figure 4B,C). Loading the surface-modified nanoparticles with paclitaxel did further increase the mean diameter (PDMS-PMOXA-GSG-Paclitaxel; 129.6 ± 1.05 nm; n = 3; one-way ANOVA, *p* < 0.05) as shown in Figure 4D. The size stability of the surface-modified nanoparticles with paclitaxel was determined over a period of 5 days. During that period, the nanoparticles swelled from 129.6 ± 1.05 nm to 143.4 ± 3.58 nm (n = 3; unpaired t-test, *p* < 0.05; Supplemental Figure S2).

A Gaussian distribution of the diameter was observed for all formulations (Figure 4B–D). These results were confirmed by electron microscopy imaging (Figure 4E), where a homogenous vesicular formulation was observable for all nanoparticles. Finally, the PDMS-PMOXA-GSG nanoparticle’s drug-loading was assessed by HPLC (Figure S3A,B, Table S3A). The loading averaged at 9.58 ± 0.67% of the deployed concentration resulting in a concentration of 19.17 ± 1.34 μg paclitaxel/mL (Table S3B).

The CAC (Supplemental Figure S4) determined for unloaded PDMS-PMOXA nanoparticles was 4.8 μg/mL (0.744.8 μM). A similar critical micelle concentration (1 μg/mL) was measured for PDMS_65_-*b*-PMOXA_14_ [31].

### 3.5. Influence of Cathepsin B on Fluorescently Labeled GSG-Surface-Modified Nanoparticles

**Figure 5:**
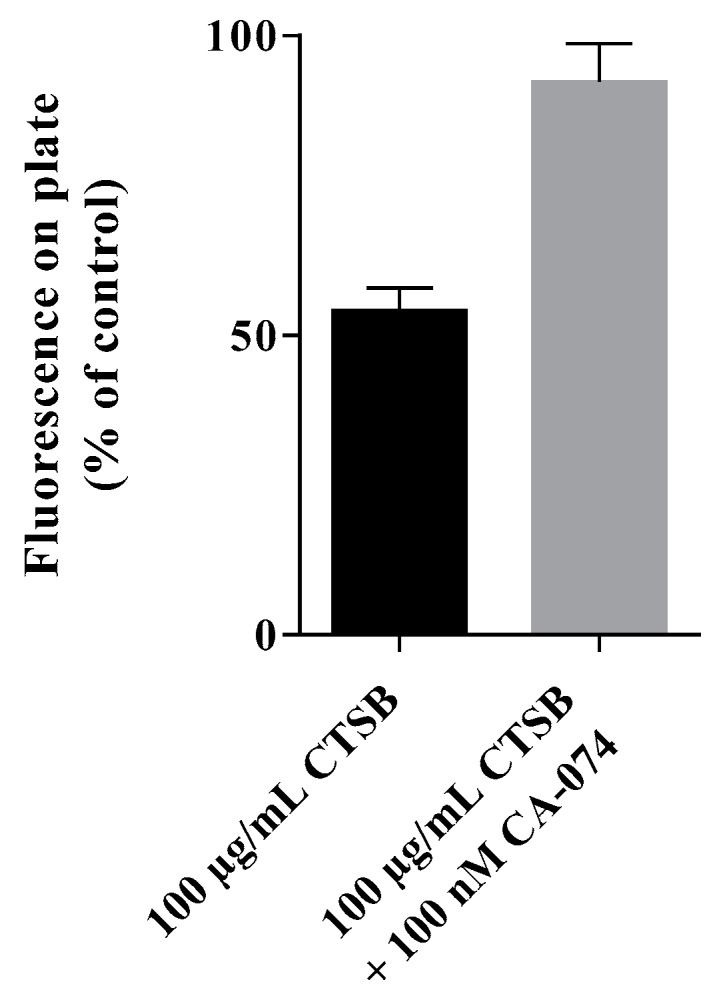
Cathepsin B triggered reduction of DiO-labeled GSG-surface-modified nanoparticles. The influence of cathepsin B was determined after covalently binding DiO-labeled GSG-surface-modified nanoparticles to the surface of a maleimide-plate. The bound nanoparticles were exposed to the enzyme in presence or absence of the CTSB-inhibitor CA-074. Data are reported as mean percent of buffer control + SD of n = 3 experiments.

DiO-labeled GSG-surface-modified nanoparticles were covalently bound to a plate surface and then exposed to cathepsin B in order to determine the influence of this enzyme on the nanoparticles. The obtained results showed a significant reduction in residual fluorescent signal after exposure to cathepsin B compared to simultaneous exposure to cathepsin B and the cathepsin B inhibitor CA-074 (Figure 5; mean % of buffer control ± SD; cathepsin B without inhibitor vs. cathepsin B with CTSB inhibitor; 54.07 ± 2.230 vs. 92.29 ± 3.723, *p* < 0.05; paired t-test), suggesting that CTSB treatment influenced the surface of the nanoparticle and thereby the DiO-loading.

### 3.6. Impact of PDMS-PMOXA-GSG-Paclitaxel Particles on Cell Viability

**Figure 6:**
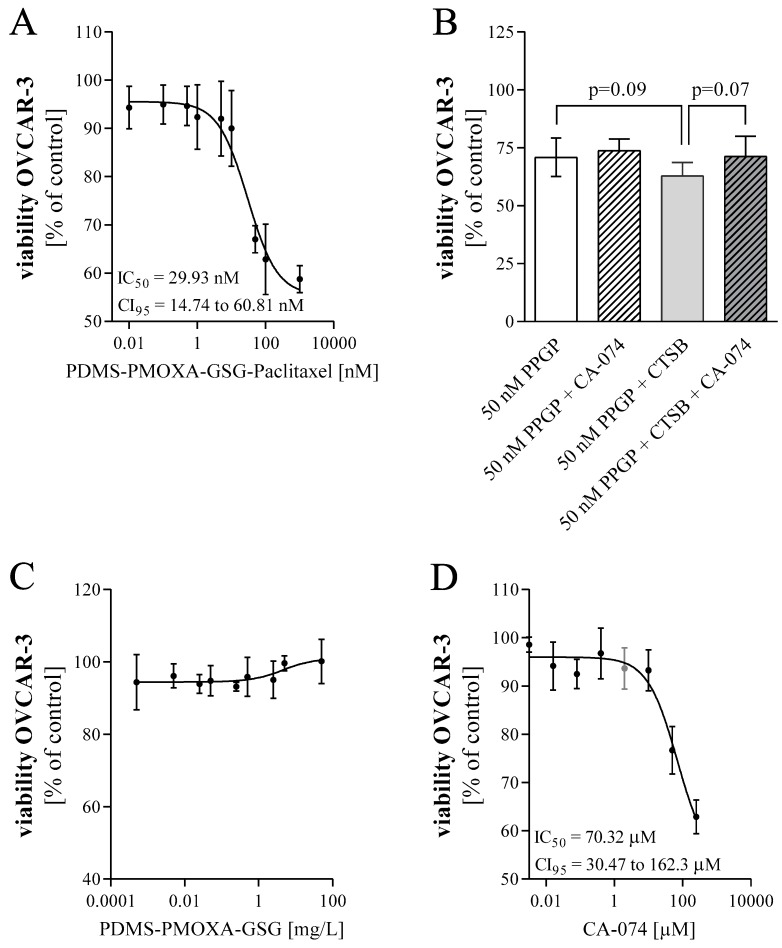
Impact of paclitaxel and paclitaxel-loaded peptide-modified particles (PPGP) on cell viability. (**A**) Cell viability of OVCAR-3 after 48 h exposure to increasing concentrations of paclitaxel-loaded cathepsin B sensitive particles. Viability was assessed using resazurin. IC50 values were calculated fitting the obtained experimental data. (**B**) Cell viability of OVCAR-3 after 48 h exposure to paclitaxel-loaded GSG-modified particles with or without inhibitor CA-074 and/or cathepsin B (CTSB). (**C**) Cell viability of unloaded peptide-modified particles was determined as control. (**D**) Cell viability of OVCAR-3 was determined after 48 h exposure to increasing concentrations of the CTSB-inhibitor CA-074. The grey data point indicates the concentration used in the previous experiment. Data are presented as mean ± SD, of n = 3 experiments each performed in technical triplicates.

In order to analyze the influence of the paclitaxel-loaded PDMS-PMOXA-GSG-particles on cell viability, OVCAR-3 cells were treated for 48 h and cell viability was determined. The quantity of paclitaxel in these particles was determined before each experiment using HPLC. As demonstrated in Figure 6A, we observed an effect of the nanoparticles on cell viability. This effect was concentration-dependent with an IC_50_ value of 29.93 nM (CI-95% 14.74–60.81 nM). As the activity of cathepsin B is suggested to modulate the release of paclitaxel from these nanoparticles, cells were treated with 50 nM paclitaxel loaded PDMS-PMOX-GSG in presence of 2 μM of the cathepsin B inhibitor CA-074 (compare Figure 6B). Even though we did not observe an increase in cell viability using 2 μM CA-074 (Mean ± SD 50 nM vs. 50 nM with CA-074; 70.94 ± 8.2% vs. 73.79 ± 4.97%) there was a decrease in viability when cells were exposed to the nanoparticles in combination with cathepsin B (62.92 ± 5.81%; one way ANOVA, *p* = 0.09;). Moreover, this effect was slightly reduced by simultaneous treatment with CA-074 (71.36 ± 8.60%; *p* = 0.07). In Figure 6C, the cell viability of unloaded peptide-modified nanoparticles showed no toxic effect on OVCAR-3 cells. Sensitivity of OVCAR-3 cells to paclitaxel was confirmed as shown in Supplemental Figure S5 (IC_50_ = 1.158 nM, CI-95% 0.39 to 3.47 nM). Finally, the CTSB inhibitor CA-074 was tested for its influence on cell viability as shown in Figure 6E (IC_50_ = 70.32μM; CI-95% 30.47–162.3 μM).

## 4. Discussion

In this study, we investigated cathepsin B digestible particles for delivery of chemotherapeutics to ovarian cancer cells. We loaded PDMS-PMOXA nanoparticles with paclitaxel and modified the particle surface with a cathepsin B cleavable peptide. A suitable in vitro model was selected and characterized before assessing the impact of the formulated nanoparticles on cellular viability.

The herein studied formulation is based on the idea to use cathepsin B as a trigger for release of a chemotherapeutic. The mode of action of this release mechanism requires increased activity of cathepsin B to ensure the triggering release close to the tumor. Comparison of expression levels of this proteolytic enzyme in healthy and malignant transformed tissue originating from various organs revealed that there is no significant difference in mRNA levels for most of the herein tested tumor entities. In this context it seems noteworthy, that the mRNA expression was assessed in a small collection of samples. Even though the sample number was low, out of the researched tumor-entities, breast cancer showed a significant reduction in cathepsin B mRNA expression compared to healthy tissue. We are not providing data on activity or protein amount. However, Berquin et al. observed decreased mRNA in RAS-transformed MCF-10A epithelial cells, while the amount and activity of the enzyme was increased compared to parenteral cells [32]. Importantly, Krepela et al. also observed increased cathepsin B activity in malignant breast carcinoma tissue [33].

The impact of tumor progression on cathepsin B mRNA expression levels was assessed considering the reported tumor stage. In cervical and endometrial cancer tissue, mRNA expression was not significantly different from healthy tissue. For ovarian carcinoma a trend towards increased mRNA expression levels was observed, especially in stage I tumors. Therefore, ovarian carcinoma was used as a model system for further investigation, supported by findings of a larger scale study on cathepsin B mRNA expression in malignant ovarian carcinoma compared to healthy tissue [34]. Interestingly, their data have shown a higher distribution of mRNA expression in malignant ovarian cancer stage I/II than in stage III/IV, which is similar to our data. Benign tumors on the other hand showed no difference in cathepsin B expression compared to healthy tissue [35]. Although mRNA expression is very interesting to discuss, it is difficult to correlate the expression level to the amount or the activity of cathepsin B due to alternative splicing variants. Concerning the cathepsin B expression, there are many regulatory steps during transcription, post-transcriptional processing, translation, post-translational processing, and trafficking [36]. The transcriptional efficiency can be influenced by different transcriptional starting points [37,38], by alternative promoters [36] and variable pre-mRNA splicing [37,39,40,41]. In tumor tissue, the mRNA splice-variant lacking exon 2 is assumed to be predominant. This splice-variant has a lower stability due to a shorter untranslated region (UTR) and it is twice as active as the mRNA including exon 2 [37]. The mRNA lacking exon 2 seems to be prone to extracellular release, especially when the expression is increased [42]. In short, the differences in the mRNA sequences are suggested to affect the stability of the mRNA, which influences translation, sorting, and activity of this enzyme [37,42].

Tumors are known for their heterogeneity in many phenotypic features including gene expression [43]. Unsurprisingly, cathepsin B appears to be non-uniformly expressed throughout the tumor but increased expressed at the border to the extracellular matrix [23]. Immunohistochemical staining of tissue sections supported the notion that there is an increased amount of cathepsin B in ovarian tumor tissue compared to healthy ovarian tissue. However, the herein reported immunohistochemistry data are only of limited validity as we are only reporting on one sample. However, they support findings of Scorilas et al. who reported similar results researching tumor tissue deriving ovarian cancer patients by immunohistochemistry [44] and of Warwas et al. describing increased cathepsin B activity in the blood serum of patients with ovarian carcinoma [35].

We characterized the ovarian cancer cell lines OVCAR-3 and OVCAR-5 for their cathepsin B expression. Both cell lines showed similar cathepsin B mRNA expression levels which is in accordance with the GEO dataset GDS4296/227961_at [45]. However, Western blot analysis of intra- and extracellular proteins showed a higher amount of cathepsin B in OVCAR-3 compared to OVCAR-5 cells. This finding was confirmed by immunofluorescent staining leading to OVCAR-3 being selected as the in vitro model system of choice for our study on CTSB-peptide-modified polymersomes.

Following our previous research on an MMP9-sensitive drug delivery system [30], a cathepsin B-sensitive drug delivery system was to be developed and investigated. For cathepsin B, there have been lesser approaches described, making it an interesting topic. Most of the researched formulations were polymer–drug conjugates with cathepsin B triggered release mechanisms [46,47,48]. These formulations use either a cathepsin B degradable polymer [46] or peptides like the herein used amino acid sequence Gly-Phe-Leu-Gly [47,48,49,50,51] for the enzyme-triggered release of the payload.

Our nanoparticles appear to be colloidally stable. However, testing the stability of the formulation over a period of 5 days revealed a significant swelling of the nanoparticles. The stability was accomplished by additional extrusion step after loading and modification. After this additional extrusion step the loading efficacy was fairly low at 9.22%, which could also depend on the used peptide.

In the herein used in vitro model system PDMS-PMOXA-GSG nanoparticles loaded with paclitaxel showed an approximately 25-fold decrease in IC_50_ compared to pure paclitaxel. This indicated a gradual release of paclitaxel during exposure with cells. A trend towards increasing cytotoxicity was observed when adding purified cathepsin B to the nanoparticles. This suggests that the added enzyme increases the digestion of the peptide surface layer and, therefore, accelerates release of payload. The cathepsin B inhibitor (CA-074) affects cell viability as it exhibits a cytotoxic effect itself with an IC_50_ of 70.32 μM. Accordingly, it is difficult to differentiate between the effect of the inhibitor and the effect of the drug paclitaxel, especially in a co-treatment. An increase in cell viability was observed after testing the nanoparticles with additional cathepsin B and inhibitor, which is in line with our expectations at a concentration of 2 μM. Treatment of cells with unloaded PDMS-PMOXA-GSG nanoparticles showed no change in cell viability.

Taken together, we report on the formulation of nanoparticles loaded with paclitaxel containing a cathepsin B digestible surface. These nanoparticles were stable with a slight tendency to swell. Furthermore, the surface-modified nanoparticles were tested in a suitable ovarian cell line for their payload release.

## Supplementary Materials

The following are available online at https://www.mdpi.com/1996-1944/12/17/2836/s1, Table S1: Cathepsin B gene expression in normal and malignant-transformed tissue. The mRNA copy-numbers were assessed by multiplex real-time PCR in different malignant-transformed tissue. Breast tissue is the only sample with statistically significant difference in cathepsin B mRNA expression, Table S2: Previous publications on cathepsin B in ovarian cancer. Literature research of publications on cathepsin B expression, protein and/or enzyme activity in ovarian cancer tissue samples, Table S3: Calculation of the encapsulation efficiency using the area under the curve. Table A contains AUC and corresponding concentration to calculate the standard curve. In Table B, the paclitaxel contained in the nanoparticles was calculated using the standard curve determined in Table A. Figure S1: Cathepsin B gene expression in normal and malignant-transformed tissue. The mRNA copy-numbers were assessed by multiplex real-time PCR in different malignant-transformed tissue. Depicted is the expression of cathepsin B mRNA in healthy and malignant tissue originating from breast (A), cervix (B), endometrium (C), and ovary (D) tissue, Figure S2: Stability of the paclitaxel-loaded surface modified polymeric nanoparticle. The hydrodynamic diameter was measured after formulation on day 1 (A) and on day 5 (B), Figure S3: Determination of the encapsulation efficiency by HPLC. Examples of HPLC UV-chromatogram recorded at wavelength 225,4 nm for the paclitaxel standard curve (A) and paclitaxel loaded PP-GSG nanoparticles (B). The retention time of paclitaxel lays between 4.8 and 4.9 mintutes, Figure S4: Critical aggregation concentration of PDMS-PMOXA. The critical aggregation concentration of PDMS-PMOXA determined by using pyrene incorporation, a hydrophobic fluorescent probe, Figure S5: Impact of paclitaxel on cell viability. Cell viability of OVCAR-3 after 48 h exposure to paclitaxel with increasing concentrations of paclitaxel. Viability was assessed using resazurin. IC_50_ values were calculated. Mean ± SD, n = 3 in technical triplicates.
