# Supplementary material for: PDMS-PMOXA-Nanoparticles Featuring a Cathepsin B-Triggered Release Mechanism
Source: Materials (Basel). 2019 Sep 3;12(17):2836. doi: 10.3390/ma12172836 (PMC6747961; doi:10.3390/ma12172836)
Supplement: Supplementary file 1 [file materials-12-02836-s001.pdf]

materials

Supplementary Materials

PDMS-PMOXA-Nanoparticles Featuring a Cathepsin B-Triggered Release Mechanism

Table S1. Cathepsin B gene expression in normal and malignant-transformed tissue. The mRNA copy-numbers were assessed by multiplex real-time PCR in different malignant-transformed tissue. Breast tissue is the only sample with statistically significant difference in cathepsin B mRNA expression.

Tissue	Healthy			Malignant			p Value
	Mean	SD	n	Mean	SD	n	
Breast	13275.80	3526.86	2	3583.20	3673.71	23	0.002
Cervix	15502.80	20071.56	4	5207.09	3474.86	9	0.144
Endometrium	2962.95	1378.70	4	3562.83	4806.66	16	0.811
Ovary	3684.62	1317.87	3	6921.85	6781.56	21	0.427

Table S2. Previous publications on cathepsin B in ovarian cancer. Literature research of publications on cathepsin B expression, protein and/or enzyme activity in ovarian cancer tissue samples.

Nr.	Year	PMID	Title	CTSB Cancer vs. Normal	Sample	Detection	Origin	Method
1	1997	9166974	Cathepsin B-Like Activity as a Serum Tumour Marker in Ovarian Carcinoma	up	T	A	H	ELISA
2	2002	12437120	Determination of Cathepsin B Expression May Offer Additional Prognostic Information for Ovarian Cancer Patients	up	T	C	H	q-PCR, ICH
3	2004	14984956	The role of cathepsin B and cystatin C in the mechanisms of invasion by ovarian cancer	up	T	C	H	WB, ICH
4	2005	16202931	Cathepsins B and D Activity and Activity Ratios in Normal Ovaries, Benign Ovarian Neoplasms, and Epithelial Ovarian Cancer	up	T	A	H	ELISA
5	2010	20727192	Increased expression of cysteine cathepsins in ovarian tissue from chickens with ovarian cancer	up	T	C	A	q-PCR, ICH
6	2014	24452274	Cystatin B is a progression marker of human epithelial ovarian tumors mediated by the TGF-β signaling pathway	up	T	C	H	q-PCR, ICH
					C = cells<br>T = tissue	A= activity<br>C = content	H = human<br>A = animal	

Table S3. Calculation of the encapsulation efficiency using the area under the curve. Table A contains AUC and corresponding concentration to calculate the standard curve. In B, the paclitaxel contained in the nanoparticles was calculated using the standard curve determined in A.

**(A) AUC**	**C (μg/mL)**	**MW (g/mol)**	**mol/L**	**μM**
9699.500	250	853.906	0.000292772	292.772
4879.200	125	853.906	0.000146386	146.386
2451.100	62.5	853.906	7.31931×10^{-5}	73.193
1221.800	31.25	853.906	3.65965×10^{-5}	36.597
601.000	15.625	853.906	1.82983×10^{-5}	18.298
402.500	7.8125	853.906	9.14913×10^{-5}	9.149
153.720	3.90625	853.906	4.57457×10^{-6}	4.575
76.560	1.953125	853.906	2.28728×10^{-6}	2.287
39.307	0.9765625	853.906	1.14364×10^{-6}	1.144
(B)				
Name	AUC	C (μg/mL)	C original (μg/mL)	%
n = 1	264.949	5.178	50.000	10.355
n = 2	241.946	4.563	50.000	9.127
n = 3	244.626	4.635	50.000	9.270

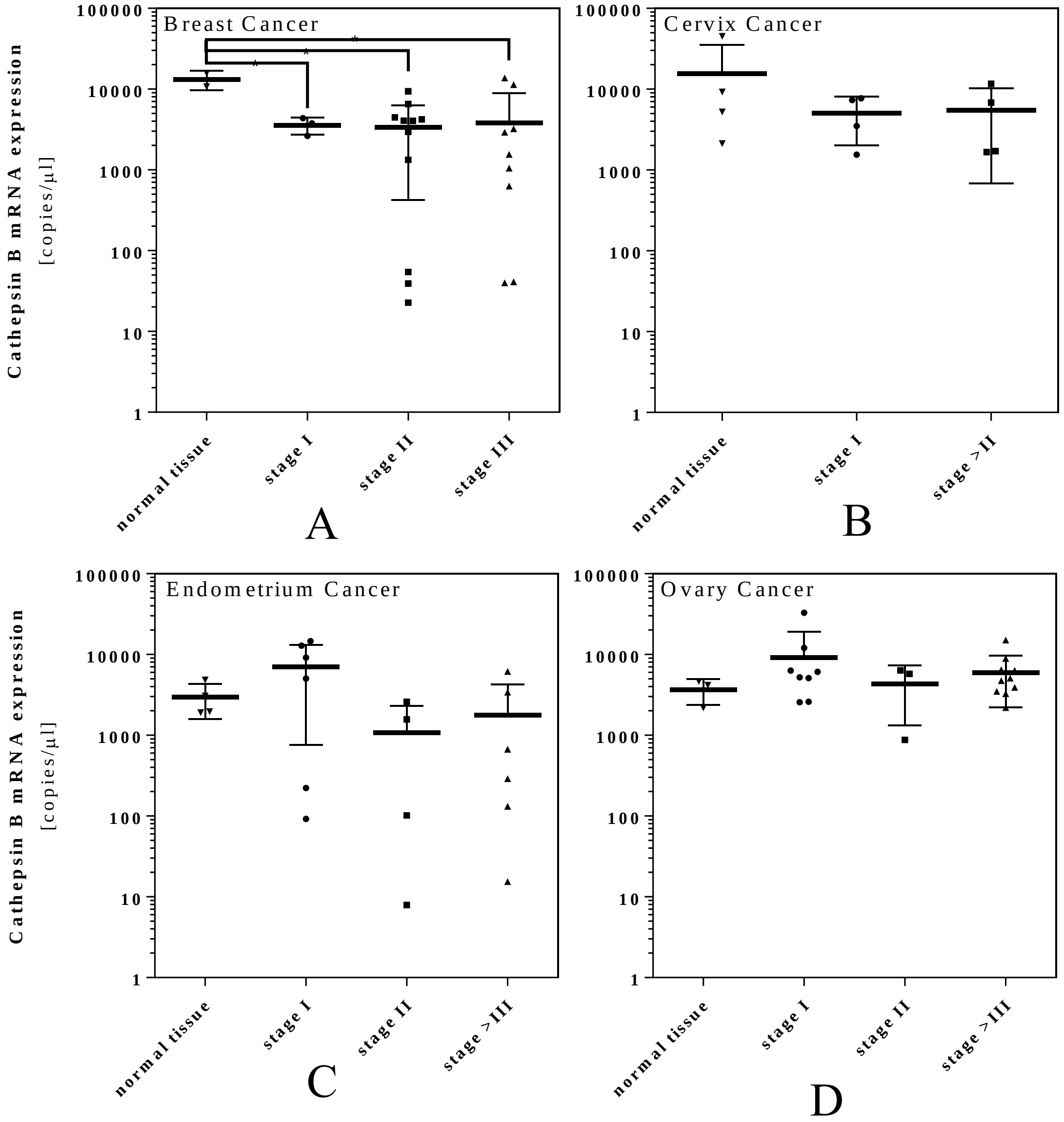

Figure S1. Cathepsin B gene expression in normal and malignant-transformed tissue. The mRNA copy-numbers were assessed by multiplex real-time PCR in different malignant-transformed tissue. Depicted is the expression of cathepsin B mRNA in healthy and malignant tissue originating from breast (**A**), cervix (**B**), endometrium (**C**), and ovary (**D**) tissue.

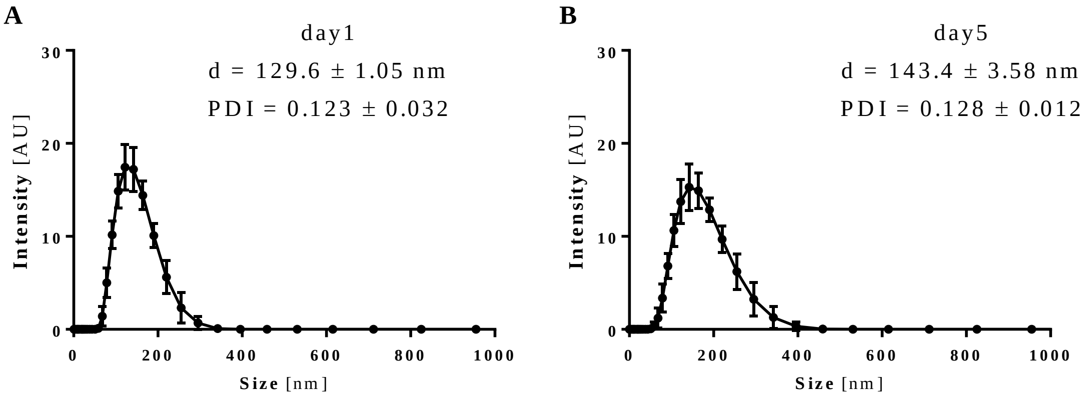

Figure S2. Stability of the paclitaxel-loaded surface modified polymeric nanoparticle. The hydrodynamic diameter was measured after formulation on day 1 (**A**) and on day 5 (**B**).

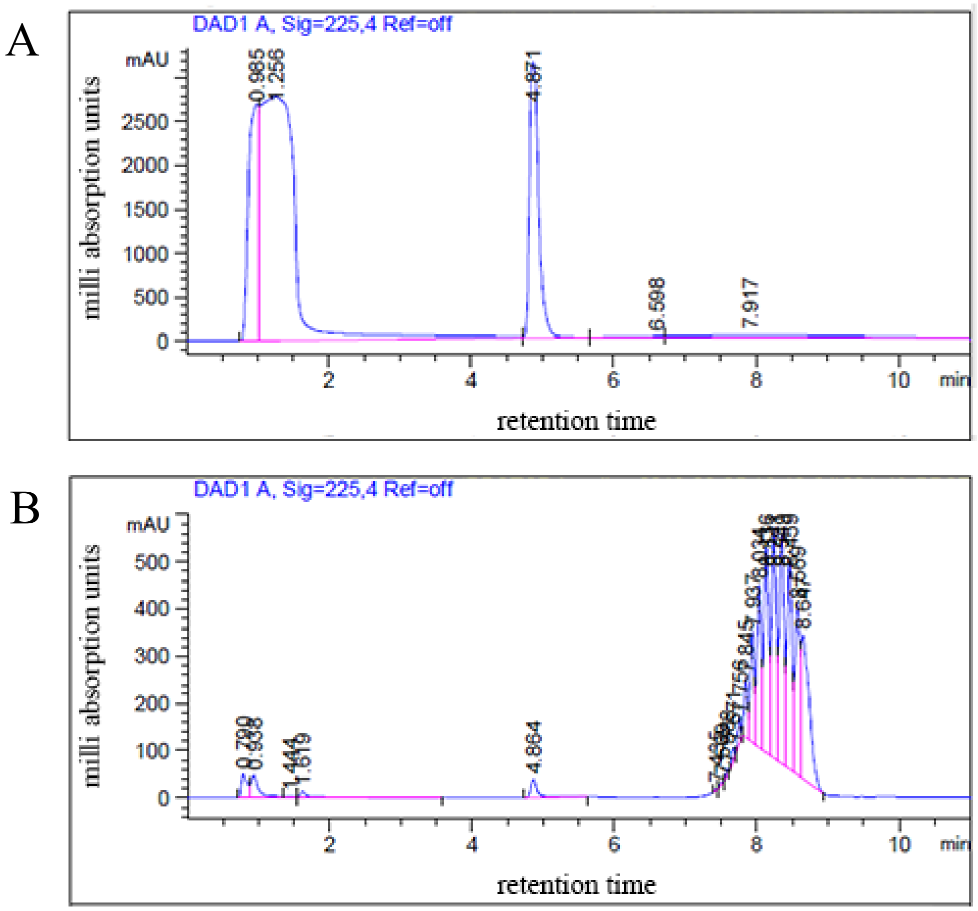

Figure S3. Determination of the encapsulation efficiency by HPLC. Examples of HPLC UV-chromatogram recorded at wavelength 225,4 nm for the paclitaxel standard curve (**A**) and paclitaxel loaded PP-GSG nanoparticles (**B**). The retention time of paclitaxel lays between 4.8 and 4.9 mintutes.

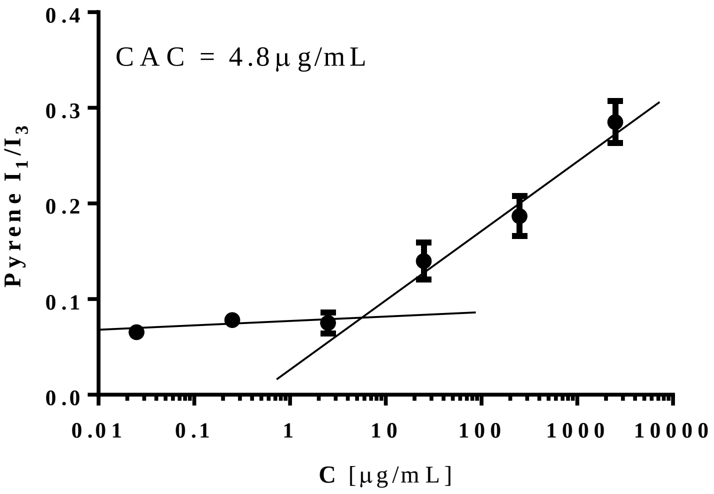

Figure S4. Critical aggregation concentration of PDMS-PMOXA. The critical aggregation concentration of PDMS-PMOXA determined by using pyrene incorporation, a hydrophobic fluorescent probe.

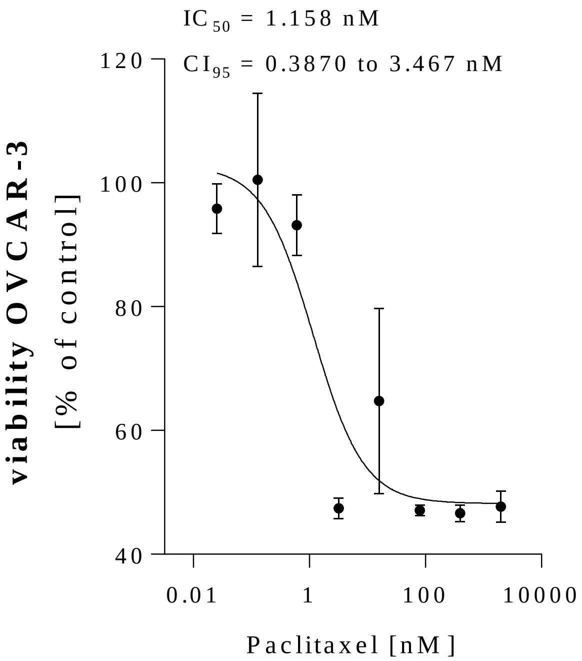

Figure S5. Impact of paclitaxel on cell viability. Cell viability of OVCAR-3 after 48 hours exposure to paclitaxel with increasing concentrations of paclitaxel. Viability was assessed using resazurin. IC_{50} values were calculated. Mean ± SD, n = 3 in technical triplicates.

© 2019 by the authors. Submitted for possible open access publication under the terms and conditions of the Creative Commons Attribution (CC BY) license (http://creativecommons.org/licenses/by/4.0/).
